# Supplementary material for: Iron and sulfide nanoparticle formation and transport in nascent hydrothermal vent plumes
Source: Nat Commun. 2019 Apr 8;10:1597. doi: 10.1038/s41467-019-09580-5 (PMC6453976; doi:10.1038/s41467-019-09580-5)
Supplement: Supplementary file 1 — Supplementary Information [file 41467_2019_9580_MOESM1_ESM.pdf]

Iron and sulfide nanoparticle formation and transport in nascent hydrothermal vent plumes

Findlay et al., 2019

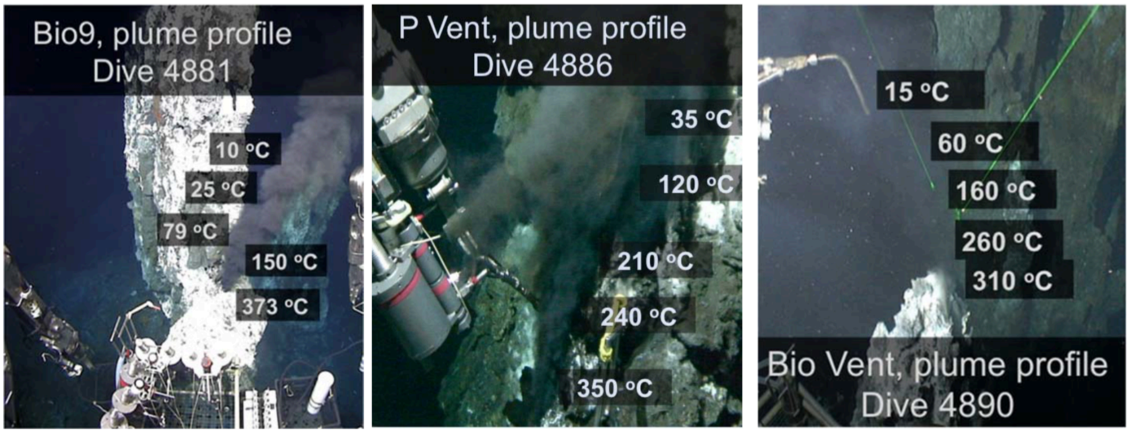

Supplementary Figure 1: Temperature profiles in the buoyant plume. All samples were taken within the first meter above the vent orifice.

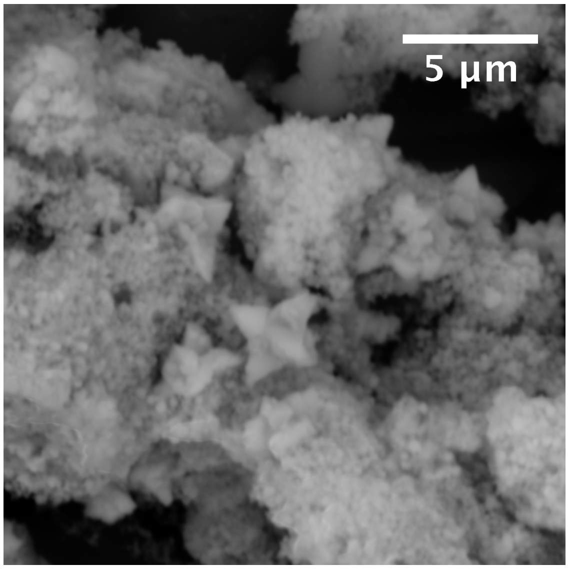

Supplementary Figure 2: Representative SEM Micrograph of mixed metal sulphide particles and nanoparticles from the end-member fluid of Bio 9. The larger, star-shaped particles have an S to Fe ratio of 2. The surrounding nanoparticles are mixed Fe, Zn and Cu sulphides. Element ratios were determined using EDS.

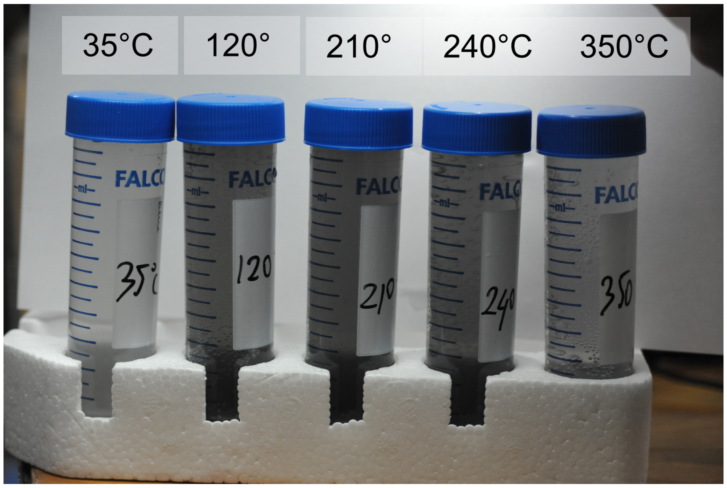

Supplementary Figure 3: Photo of plume samples from the plume at P Vent, showing the visual difference in particle density over the temperature gradient.

					filtered (0.2 µm)				unfiltered					filtered (0.2 µm)		unfiltered					
Vent	Distance from orifice (cm)	[Mg] (µM)	T (°C)	pH	$[Fe^{2+}]$ (µM)	std dev	$[Fe_{Total}]$ (µM)	std dev	$[Fe^{2+}]$ (µM)	std dev	$[Fe_{Total}]$ (µM)	std dev	% Fe < 0,2 µm	AVS (µM)	CRS (µM)	AVS (µM)	CRS (µM)	total sulphide (µM)	CRS as % total sulphide	filtered CRS/Fe_{py}	S:Fe
Bio9	0	3.40	373	3.13	1357	*17*	1330	3	1328	*8*	1346	15	102	4887	246	5966	819	6784	3.6	--	5
	2	43.7	150	4.87	213	*7*	248	9	279	*17*	280	48	76	746	216	1120	454	1574	13.7	5.0	6
	5	52.2	79	5.31	116	*7*	124	6	132	*6*	155	4	88	408	58	306	196	501	11.6	**2.5**	3
	10	51.4	25	5.58	75	*2*	86	3	91	*1*	115	4	82	165	22	335	112	447	5.0	**2.5**	4
	20	51.1	10	6.28	19	*1*	22	1	22	*1*	27	4	84	0	0	2	2	4	0.0	0.0	0.1
P Vent	0	5.3	350	3.27	1034	14	1048	40	1048	13	1065	9	99	4735	204	4595	1065	5659	3.6	--	5
	5	25.9	240	3.93	498	6	518	5	592	7	615	17	84	3142	256	2682	1438	4121	6.2	13.0	7
	10	28.3	210	4.01	321	4	339	7	425	10	430	6	76	2794	441	2464	1154	3619	12.2	24.6	8
	20	46.0	120	4.87	230	14	235	3	294	4	301	8	78	1569	72	1624	487	2111	3.4	5.5	7
	100	51.5	35	5.73	52	2	67	7	62	2	73	3	83	94	29	178	94	272	10.8	**1.9**	4
Bio Vent	0	11.5	310	3.77	301	2	283	4	310	6	315	9	97	4523	63	5333	198	5531	1.1	--	18
	2	20.4	260	4.10	186	4	181	13	216	5	308	19	86	3521	134	3957	293	4250	3.1	--	14
	5	32.4	160	4.34	151	18	124	10	161	6	173	17	94	2735	89	3368	319	3688	2.4	--	21
	50	47.1	60	4.77	87	1	94	6	88	9	99	3	98	1506	62	1755	138	1892	3.3	--	19
	100	54.0	10	5.79	15	1	24	3	15	0	31	1	98	123	18	142	30	172	10.4	**2.0**	6

Supplementary Table 1: Total and filtered Fe and sulphide (AVS and CRS) concentrations for each sample. CRS/Fe_{py} represents the ratio between filtered CRS and nanoparticulate Fe as determined from Fe analyses (for more details, please refer to the main text). If all CRS < 0.2 µm is pyrite, the ratio should equal 2.

Location	Temperature (˚C)	Minerals
Bio 9	373	Pyrite, Chalcopyrite, Sphalerite, nano ZnS
	150	α-Sulfur, Barite
	79	α-Sulfur, Sphalerite
	25	α-Sulfur
	10	N/A
P-vent	350	Pyrite, Anhydrite, Chalcopyrite/Isocubanite, Gordaite, nano ZnS
	240	α-Sulfur, Sphalerite, Pyrite
	210	α-Sulfur
	120	α-Sulfur
	35	N/A
Biovent	310	α-Sulfur, Chalcopyrite/Isocubanite, Sphalerite, nano ZnS
	260	α-Sulfur, Sphalerite, Gordaite, nano ZnS
	160	α-Sulfur, Gordaite, Sphalerite, Barite, nano ZnS
	60	Gordaite, α-Sulfur
	10	N/A

Supplementary Table 2: Bulk mineralogy in samples from the end-member and within the first meter of buoyant plume of each vent.
